# Comparative Transcriptome Profiling of Human and Pig Intestinal Epithelial Cells after Porcine Deltacoronavirus Infection

**DOI:** 10.3390/v13020292

**Published:** 2021-02-13

**Authors:** Diana Cruz-Pulido, Patricia A. Boley, Wilberforce Zachary Ouma, Moyasar A. Alhamo, Linda J. Saif, Scott P. Kenney

**Affiliations:** 1 Department of Veterinary Preventive Medicine, Food Animal Health Research Program (FAHRP), Wooster, OH 44691, USA; cruz-pulido.1@osu.edu (D.C.-P.); boley.28@osu.edu (P.A.B.); abdulhameed.1@osu.edu (M.A.A.); saif.2@osu.edu (L.J.S.); 2 The Ohio Supercomputer Center (OSC), Columbus, OH 43212, USA; wouma@osc.edu

**Keywords:** porcine deltacoronavirus, PDCoV, RNAseq, zoonosis, coronavirus, cross-species transmission, differentially expressed host genes, cytokines, interferons

## Abstract

Porcine deltacoronavirus (PDCoV) is an emerging infectious disease of swine with zoonotic potential. Phylogenetic analysis suggests that PDCoV originated recently from a host-switching event between birds and mammals. Little is known about how PDCoV interacts with its differing hosts. Human-derived cell lines are susceptible to PDCoV infection. Herein, we compare the gene expression profiles of an established host swine cells to potential emerging host human cells after infection with PDCoV. Cell lines derived from intestinal lineages were used to reproduce the primary sites of viral infection in the host. Porcine intestinal epithelial cells (IPEC-J2) and human intestinal epithelial cells (HIEC) were infected with PDCoV. RNA-sequencing was performed on total RNA extracted from infected cells. Human cells exhibited a more pronounced response to PDCoV infection in comparison to porcine cells with more differentially expressed genes (DEGs) in human, 7486, in comparison to pig cells, 1134. On the transcriptional level, the adoptive host human cells exhibited more DEGs in response to PDCoV infection in comparison to the primary pig host cells, where different types of cytokines can control PDCoV replication and virus production. Key immune-associated DEGs and signaling pathways are shared between human and pig cells during PDCoV infection. These included genes related to the NF-kappa-B transcription factor family, the interferon (IFN) family, the protein-kinase family, and signaling pathways such as the apoptosis signaling pathway, JAK-STAT signaling pathway, inflammation/cytokine–cytokine receptor signaling pathway. MAP4K4 was unique in up-regulated DEGs in humans in the apoptosis signaling pathway. While similarities exist between human and pig cells in many pathways, our research suggests that the adaptation of PDCoV to the porcine host required the ability to down-regulate many response pathways including the interferon pathway. Our findings provide an important foundation that contributes to an understanding of the mechanisms of PDCoV infection across different hosts. To our knowledge, this is the first report of transcriptome analysis of human cells infected by PDCoV.

## 1. Introduction

Coronaviruses (CoVs) are an ancient lineage of viruses with their most recent common ancestor potentially occurring millions of years ago and evolving over time with their hosts [1]. Phylogenetically, CoVs are classified into four genera: *Alpha*-, *Beta*-, *Gamma*- and *Delta-coronavirus* [2,3]. The alpha and beta CoVs are thought to primarily derive from bat lineages, whereas gamma and delta CoVs primarily come from avian hosts [3,4]. The most notorious of these spillover events include members of the *Betacoronavirus* genus. Severe acute respiratory syndrome (SARS) CoV, Middle East respiratory syndrome (MERS) CoV, and SARS-CoV-2 are known to be zoonotic, causing lethal respiratory infections in humans [5,6]. Each of these viruses found their way into humans via a spillover event involving bats as the likely primary host, followed by an intermediate host, prior to gaining the ability to infect humans. In 2002, SARS transited from horseshoe bats (*Rhinolophus genus*) through palm civet cats (*Paguma lavata*) before infecting humans in a live animal market in Guangzhou, China [7]. The virus spread rapidly to thousands of humans in numerous countries worldwide and disappeared after strict implementation of quarantine measures and the culling of civet cats in the wet markets [8]. MERS was first isolated in a human pneumonia patient in 2012. Retrospective studies revealed that MERS-CoV infected dromedary camels as early as 1983 [9], while CoVs similar to MERS strains in humans were found in bats [10]. The most recent addition to CoV spillover is the emergence of SARS-CoV-2 in December of 2019 that has since become a global pandemic [11,12]. While the origins of SARS-CoV-2 are unclear, sequences with high similarity have been found in bats, with pangolins suggested as a possible intermediate host [13,14,15].

The devastating effects of CoVs are not limited to humans but also occur in livestock populations, particularly pigs. Transmissible gastroenteritis virus (TGEV), porcine epidemic diarrhea virus (PEDV), porcine deltacoronavirus (PDCoV), and swine acute diarrhea syndrome (SADS) have all emerged from reservoir hosts and cause enteric disease in pigs, in some cases on a global scale [16,17]. Understanding factors driving spillover events between species is critical for preventing further CoV spillover into agriculturally important animals and humans.

The *Deltacoronavirus* genus contains primarily avian CoV pathogens of songbirds including bulbul coronavirus HKU11, thrush coronavirus HKU12, and munia coronavirus HKU13 [4]. The identification of *deltacoronavirus* in an Asian leopard cat (*Prionailurus bengalensis*)** and HKU15 (PDCoV) in pigs are the first known mammalian members of the *Deltacoronavirus* genus, and in pigs, they also cause morbidity. PDCoV’s high sequence homology with sparrow DCoV suggests it may be an early spillover event or recently adapted from an avian CoV that infects mammals [4,18]. Phylogenetic analysis suggests that PDCoV originated relatively recently, within the last several centuries, from a host-switching event between birds and mammals [4,19].

PDCoV belongs to the *Deltacoronavirus* genus of the Coronaviridae family [4,20]. It is a single-stranded, enveloped, positive-sense RNA virus [21] with a genome of approximately 25 kb in length. The genome encodes the following: open reading frame 1a/1b that occupies about two thirds of the viral genome and produces the viral replication machinery; the structural [spike (S), envelope (E), membrane (M), nucleocapsid (N)] and nonstructural (NS6/NS7) proteins [22,23]. PDCoV is an emerging global infectious disease of the swine industry causing mortality in up to 40% of infected neonatal pigs [24,25]. It was first reported in Hong Kong, China in 2012 and isolated from clinical cases of major diarrhea outbreaks in young pigs in the United States in 2014 [26]. This virus was reportedly associated with clinical signs of acute watery diarrhea in sows and piglets [20,27].There are currently no treatments or commercially available vaccines for PDCoV [5,28]. Although diagnostic tests such as one-step quantitative reverse transcription PCR (RT-qPCR) and enzyme linked immunosorbent assay (ELISA) have been established for PDCoV, effective treatments and control measures for swine PDCoV infections are lacking due to the unknown mechanisms of PDCoV infection [29,30]. CoVs have evolved several strategies for efficient replication in the host. Some strategies involve engagement of the apoptotic machinery for efficient viral infection, and the ability to escape the innate immune response by impeding the activation of transcription factors IRF3 and NF-kB, both of which are involved in the RIG-I signaling pathway, disrupting IFN-B production [28,31,32].

An increase in the number of coronaviruses that have been discovered—and availability of their genome sequences—provides opportunity for performing genomics and bioinformatics analyses on this family of viruses [2]. Little is known about how PDCoV interacts with its differing hosts. Recently, Li W. et al. showed that PDCoV can infect cells from an exceptionally diverse range of species by binding to an interspecies conserved domain of APN [19]. APN, also known as CD13 [33], is shared by many coronaviruses and even other pathogens such as *E. coli* F4 [34,35]. Viruses gain an evolutionary advantage by utilizing phylogenetically conserved receptors, resulting in host switching and virus speciation [36]. Jiang et al. indicated that the innate immune-associated genes and signaling pathways in PK-15 cells could be affected by PDCoV infection [28]. Integrating bioinformatics methods across host species enables the prediction of viral evolution and the associated outcome of viral infection in patients and subsequent adjustments of therapeutic treatments [37]. Consequently, we hypothesize that gene expression may be different depending on the cell type and species in which the infection occurs. We test this hypothesis by performing RNA-seq transcriptome profiling of intestinal epithelial human and swine cells infected by PDCoV.

To explore and compare transcriptome profiles of human-versus-porcine cell lines, this study first investigated whether human intestinal epithelial cells (HIECs) are susceptible to infection with cell-culture-adapted PDCoV. Next, we identified differentially expressed human and swine epithelial cell genes in response to PDCoV. We identified common differentially expressed genes (DEGs) and signaling pathways between human and pig cells. To our knowledge, this is the first report of transcriptome analysis of human cells infected by PDCoV in comparison to cells from its natural host.

## 2. Materials and Methods

### 2.1. Virus

The PDCoV OH-FD22 p101 cell-culture-adapted virus was previously isolated from small intestinal contents of a diarrheic pig from Ohio using LLC porcine kidney (LLC-PK) cell cultures [20,27]. The infectious titer as determined by tissue culture infectious dose 50 (TCID50) [38,39] and plaque assay was 7 × 10^7^/mL (1 ×10^7^ PFU/mL).

### 2.2. Intestinal Epithelial Swine (IPEC-J2), Human Intestinal Epithelial Cells (HIEC), and PDCoV Infection

IPEC-J2 [40] and HIEC (HIEC-6 ATCC CRL-3266^TM^) cells were provided by Linda Saif, Food Animal Health Research Program, The Ohio State University, Wooster, OH, USA. IPEC-J2 cells (passage 16) were cultured in Dulbecco’s modified eagle medium/F12 (DMEM/F12) (Thermo Fisher, Waltham, MA, USA) supplemented with 20 mM 4-(2-hydroxyethyl)-1-piperazineethanesulfonic acid (HEPES), 25 mL (5% *v*/*v*) fetal bovine serum (FBS), 5 mL 1000 U/mL penicillin/streptomycin (1%) (Thermo Fisher), 5 mL insulin-transferrin-sodium selenite (100X or 0.1722 mM-0.006875 mM-0.0038728 mM, respectively) (Thermo Fisher), and 5 ng/mL of human epidermal growth factor, EGF (BioVision) [41]. HIEC cells (passage 3) were maintained on type I collagen-coated culture dishes in Opti-MEM reduced serum medium (Thermo Fisher) supplemented with HEPES (20 mM) (Thermo Fisher), GlutaMAX (10 mM) (Thermo Fisher), 20 mL FBS (4% *v*/*v*) and 10 ng/mL of human epidermal growth factor, EGF (BioVision, Milpitas, CA, USA). PDCoV OH-FD22 [20] was used to infect the cells at a multiplicity of infection (MOI) of 1. To infect, cells were rinsed with maintenance medium (50 mL of advanced MEM supplemented with 0.5 mL of 100X anti–anti (1% *v*/*v*)), 0.5 mL (1% *v*/*v*) of 100X MEM nonessential amino acids (NEAA), and 0.5 mL of HEPES (1% *v*/*v*)), and the virus was added and allowed to adsorb for one hour in the presence of 0.25% Trypsin (HIEC cells) and 0.05% trypsin (IPEC cells). Cells were harvested for RNA extraction at 24 h postinfection (hpi) as described below.

### 2.3. Immunofluorescent Staining (IF) for the Detection of PDCoV Antigen in HIEC Cells

At 24 hpi HIEC cells (passage 3) either infected with PDCoV or mock-infected were rinsed with phosphate-buffered saline (PBS) and fixed with 2 mL paraformaldehyde (4%) solution in phosphate-buffered saline (PBS) for 30 min. Two mL (0.2% *v*/*v*) Triton X-100 (Millipore Sigma, Burlington, MA, USA) was used as a permeabilization agent for 15 min at room temperature. Fixed cells were blocked using BlockerTM Universal Blocking Solution (Thermo Fisher, Waltham, MA, USA) for 30 min followed by incubation for 1 h at 37 °C with the primary antibody (Mouse anti-N monoclonal antibody (1:2500) kindly supplied by Dr. Steven Lawson at South Dakota State University). Cells were washed three times, and the staining was completed by adding the secondary antibody (Alexa Fluor 488-conjugated goat α-mouse antibody (1:400) (Thermo Fisher, Waltham, MA, USA). Nuclei were visualized using 4′,6-diamidino-2-phenylindole (DAPI) (Thermo Fisher, Waltham, MA, USA). Cells were observed using an Olympus IX-7 fluorescent microscope.

### 2.4. RNA Extraction and Quality Control

RNA was extracted using a GenCatch total RNA miniprep kit (Epoch Life Science, Sugar Land, TX, USA) following the manufacturer’s instructions. In order to remove all traces of DNA, RNA samples were treated with DNase I using TURBO DNAase (Thermo Fisher, Waltham, MA, USA). IPEC and HIEC cells served as the host uninfected control samples (6 samples—control). Three replicates were used per sample for a total of 12 samples. RNA quality was assessed by using TapeStation Analysis Software A.02.02 (Agilent, Santa Clara, CA, USA). According to NEBNext^®^ Ultra II Directional RNA Library Prep Kit for Illumina, samples with RNA integrity (RINs) equal to 2 to 7 or greater than 7 were selected for library preparation and sequencing [42].

### 2.5. Library Preparation and Sequencing

The whole transcriptome RNA was enriched by depleting ribosomal RNA (rRNA) using a NEBNext rRNA depletion kit (New England Biolabs (NEB, Ipswich, MA, USA)). Input RNA concentrations, fragmentation conditions, and PCR cycles for intact and degraded RNA were established following the manufacturer’s protocol. RNA and cDNA samples were purified by using DNA purification SPRI magnetic beads (abm good, Richmond, BC, Canada). Completed libraries were quantified by using the Qubit assay kit (Thermo Fisher) and analyzed via TapeStation Analysis Software (Agilent) and Bioanalyzer to determine the library size. Libraries were pooled and sequenced using Illumina Hiseq Platform PE150 (Novogene, Sacramento, CA, USA).

### 2.6. Data Preprocessing and Alignment

Raw sequence reads were first subjected to a quality check that involved removal of adapter sequences by using FastQC [43] and BBMap [44]. Reads were aligned to *Homo sapiens* GRCh38 genome release 97 (ftp.ensembl.org/pub/release-97/fasta/homo_sapiens/dna/ (accessed on 13 January 2020)) and *Sus scrofa* 11.1 genome release 97 (ftp://ftp.ensembl.org/pub/release-97/fasta/sus_scrofa/dna/ (accessed on 10 February 2020)) using the Rsubread aligner [45]. Raw sequence reads are available in SRA (Bioproject No. PRJNA690955). Computer code is available in GitHub (https://github.com/Diana-Ouma/Comparative-transcriptome-analysis (accessed on 30 December 2020)).

### 2.7. Expression Data Preprocessing

Gene expression counts were identified from the alignment files in BAM format using Rsubread [45]. Raw count data were transformed to counts per million (CPM) and log-CPM using EdgeR [45]. Genes that were not expressed in any biologically significant levels (CPM less than 1) were discarded. Expression values were normalized using the trimmed mean of M-values (TMM) normalization [46].

### 2.8. Differential Expression

Differential expression analysis was performed between infected and mock samples in each cell line. In brief, dispersion of each gene was first estimated, followed by bit-fitting generalized linear models (GLM) on the expression dataset. Differential expression was tested using a quasi-likelihood (QL) F-test method. Analyses were performed using the Limma and EdgeR statistical packages on the R programming environment [45]. Bonferroni–Hochberg adjusted *p*-value cut-off of 0.05 and log fold change of 1 (FDR ≤ 0.05) were employed in identifying statistically significant DEGs between uninfected control and the infected cell lines.

### 2.9. GO Function and KEGG Pathway Enrichment Analysis

Function classification and enrichment analysis of DEGs were performed using KEGG gene set enrichment analysis available in the R package ClusterProfiler, as well as using DAVID [47] and PANTHER 15.0 software [48]. Pathway enrichment was analyzed based on the KEGG database [49]. KEGG pathways with *p*-values < 0.05 were considered to be significantly enriched.

Intersections of common up-regulated and down-regulated DEGs across pathways and between species were represented by Venn Diagrams using the draw-custom-Venn Diagram tool [50] and orthologous genes between human and pig cells were predicted using PANTHER 15.0 [48] and protein BLAST from NCBI [51] with the following parameters: E-values greater than 10-6, identity percentages greater than 30%, and less than 97%, and query coverage greater than 95%.

### 2.10. Selection of Genes for RT-qPCR Validation and Primer Design

We selected glyceraldehyde 3-phosphate dehydrogenase (GAPDH) as a reference gene and measured the relative gene expression levels for several target candidate genes by RT-qPCR assay. Gene-expression stability of GAPDH was evaluated in porcine IPEC-1 cells [52]. The primer pairs were designed using either primer quest tool (Integrated DNA Technologies) or real-time PCR Primer and probes design tool (Genscript, Piscataway, NJ, USA) using full-length genomic sequences from each gene found in GenBank. Details of the primer sequences are provided in Supplementary Materials Table S1.

### 2.11. Reverse-Transcription Quantitative Polymerase Chain Reaction and Viral RNA Titers (RT-qPCR)

Reference RNA from the same samples used in RNA-seq was utilized to generate cDNA using the iScript™ Advanced cDNA Kit (Bio-Rad, Hercules, CA, USA), as directed, to generate cDNA at 50 ng/uL. Prior to conducting RT-qPCR, conventional PCR was conducted for each set of primers to determine optimal conditions, and the products were visualized on agarose gel before proceeding.

RT-qPCR for each transcript was carried out in opaque white 96 well plates using PowerUp™ SYBR™ Green Master Mix (Thermo Fisher), as directed. A standard curve was generated for each gene before complete testing commenced. RT-qPCR was conducted on a Mastercycler^®^ Realplex real-time PCR system (Eppendorf, Enfield, CT, USA). All samples were tested in triplicate, and data were calculated using the double delta Ct method [53].

Viral RNA titers were determined by rRT-PCR (QIAGEN, Valencia, CA, USA), in brief, an amplified 541-bp fragment of the M gene that covered the qRT-PCR-amplified fragment. Primers (5′-CGCGTAATCGTGTGATCTATGT-3′ and 5′-CCGGCCTTTGAAGTGGTTAT-3′) were designed according to the sequence of a U.S. strain, Illinois121/2014 (GenBank accession no. KJ481931). The PCR products were purified using a QIAquick PCR purification kit (Qiagen Inc., Valencia, CA, USA), sequenced, and then used as the template to construct a qRT-PCR standard curve. The detection limit of the rRT-PCR was 10 genomic equivalents (GEs)/reaction, which corresponded to 4.6 log_10_ GE/mL of PDCoV.

## 3. Results

### 3.1. HIEC Cells Are Susceptible to PDCoV Infection

**Figure 1:**
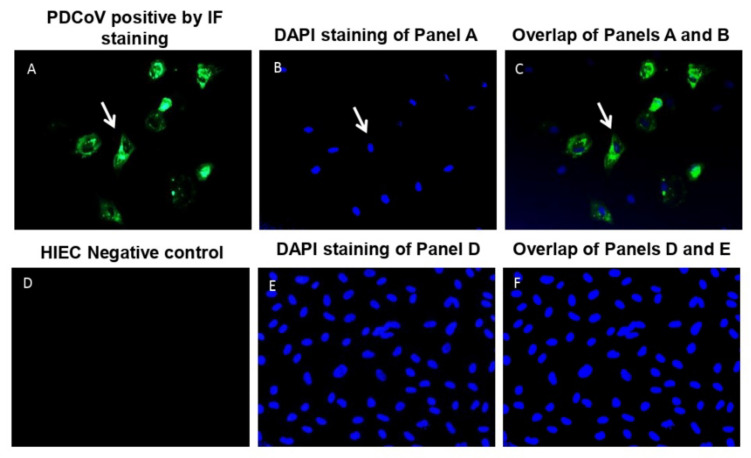
IF staining of the inoculated HIEC cells at 24 hpi. (**A**) IF-stained positive (green fluorescence) for PDCoV antigen. (**B**) Blue-fluorescent 4′,6-diamidino-2-phenylindole dihydrochloride (DAPI) staining of Panel A to stain nuclear DNA. (**C**) Overlay of panels A and B. The arrowheads indicated cells positive for PDCoV antigen. (**D**) IF staining of PDCoV-un-inoculated (0.05% Trypsin). (**E**) DAPI staining of Panel D. (**F**) Overlay of Panels D and E. Fluorescent images were taken at 300× total magnification.

HIEC cells are a normal nonimmortalized, non-transformed human intestinal crypt cell derived from fetal immature small intestines [54]. These cells have been useful in studying human crypt-cell functions such as proliferation, apoptosis, cell-matrix interactions, metabolism, and inflammatory response [54]. We observed that HIEC cells are susceptible to PDCoV infection, as confirmed at 24 hpi via immunofluorescent staining (IF) (Figure 1A–C), compared to mock-infected cells (Figure 1D–F). Additionally, HIEC cells appear susceptible to PDCoV-mediated cell death as infected cells were reduced in number at 24 hpi and were almost completely killed at 48 hpi compared to mock. We further confirmed that IPEC-J2 cells were susceptible to PDCoV infection as previously reported [41], and equivalent positive IF signals to that of PDCoV-infected HIECs at 24 hpi were observed (Figure S1).

### 3.2. PDCoV Infection Results in More Differentially Expressed Genes in Human Cells Compared to Pig Cells

More than 23 million processed reads were employed in uncovering the global gene expression profile of HIEC and IPEC cells after PDCoV infection. The reads showed total mapping rates ranging from 81.59 to 95.83% (Table 1).

**Figure 2:**
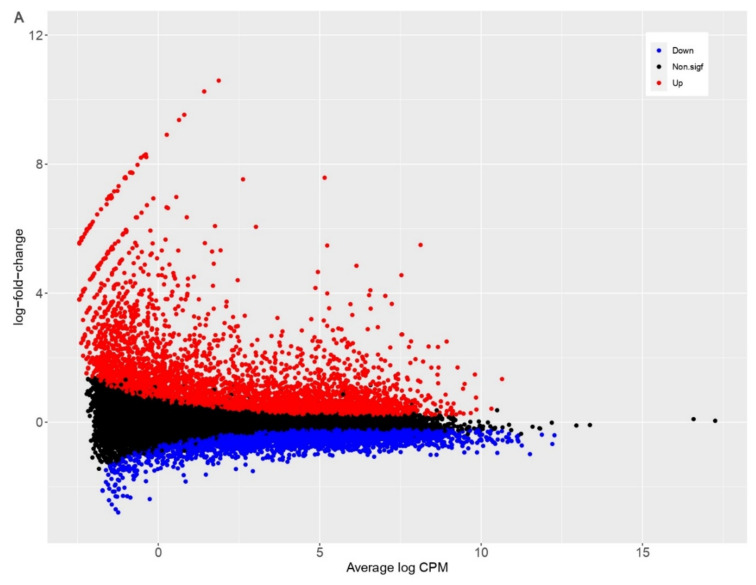

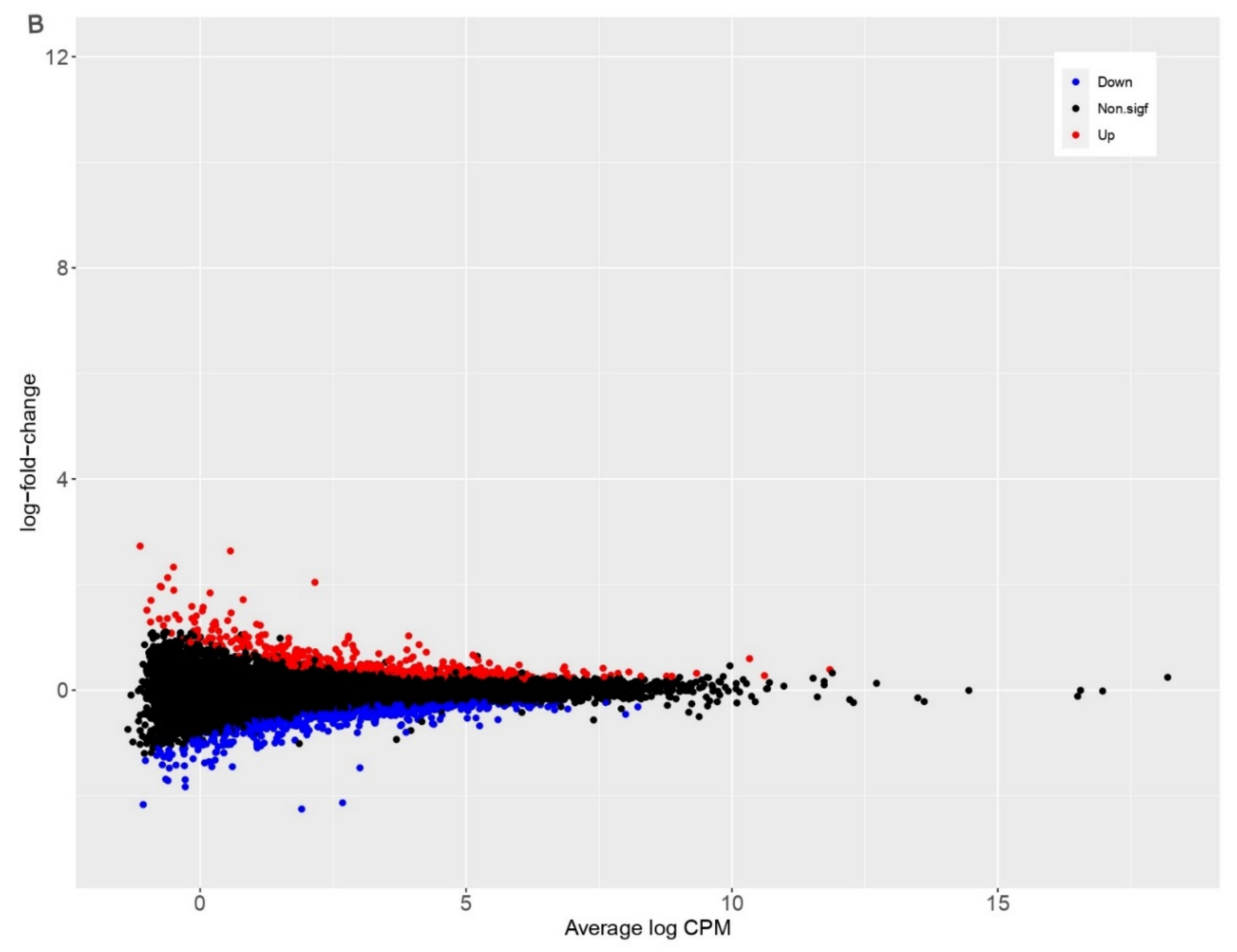
Differentially expressed genes (DEGs) in *H. sapiens* (human intestinal epithelial cells (HIEC)) (**A**) and *S. scrofa* (Porcine intestinal epithelial cells (IPEC)) (**B**) at 24 hpi vs non-infected cells. DEGs up-regulated are represented by red dots, DEGs down-regulated are represented by blue dots, and genes with no significant differences in expression are represented by black dots.

Differential expression analysis of HIEC cells resulted in identification of 7486 DEGs. Of these, 4011 genes were up-regulated, and 3475 genes down-regulated upon PDCoV infection (Table 2, Figure 2A).

Conversely, infection of IPEC cells resulted in identification of 1134 DEGs, wherein 542 genes were up-regulated, and 592 genes down-regulated, at 24 hpi (Table 2, Figure 2B). These results show that more genes are up-regulated in human cells compared to pig cells upon PDCoV infection (Figure 2).

**Figure 3:**
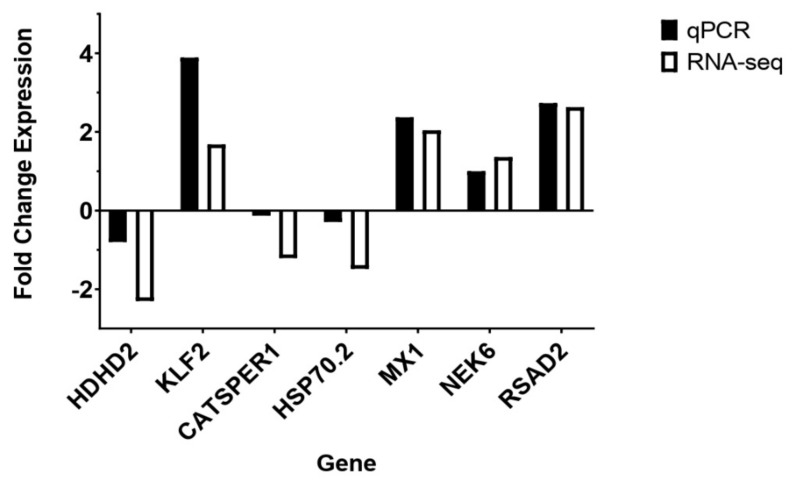
Validation of DEGs from RNA-seq in HIEC and IPEC cells.

**Figure 4:**
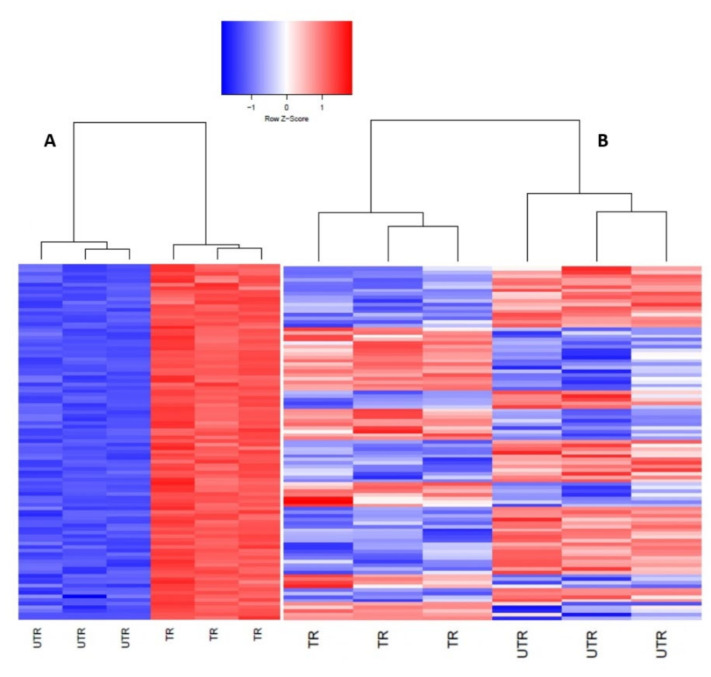
Hierarchically clustered heatmap of the top 100 genes that were differentially expressed in the PDCoV-infected HIEC cells (humans) (**A**) and the PDCoV-infected IPEC cells (pigs) (**B**) at 24 hpi compared to the non-infected cells (*p* < 0.05). Each line above represents grouping of samples at different levels. Up-regulated genes were represented in red, and down-regulated genes were represented in blue. UTR, non-infected cells; TR, infected cells.

To validate RNA-Seq results, seven transcripts (three down-regulated and four up-regulated pig and human genes) were randomly selected for RT-qPCR. Results from RT-qPCR showed that the relative expression of these genes was similar to our RNA sequencing results (Table 3, Figure 3). Next, we set out to visualize expression profiles of the top 100 DEGs using a hierarchically clustered heatmap. We observed that unlike genes in pig cells, the top 100 DEGs in human cells were all up-regulated upon infection (Figure 4A), while the top 100 DEGs in pig cells comprised genes that were either up- or down-regulated upon infection (Figure 4B). Thus, a group of genes exhibiting the highest magnitude of differential expression upon infection in human cells was up-regulated, while the group of genes with the highest magnitude of differential expression in pig cells comprised genes that were both up- and down-regulated, upon PDCoV infection.

### 3.3. Common Pathways and Genes Are Affected in the Immune Associated Response to PDCoV Infection in Human and Pig Cells

**Figure 5:**
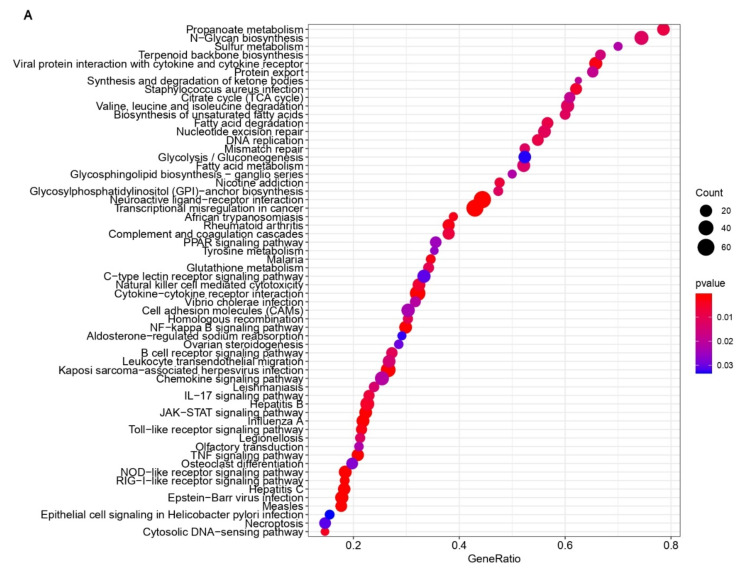

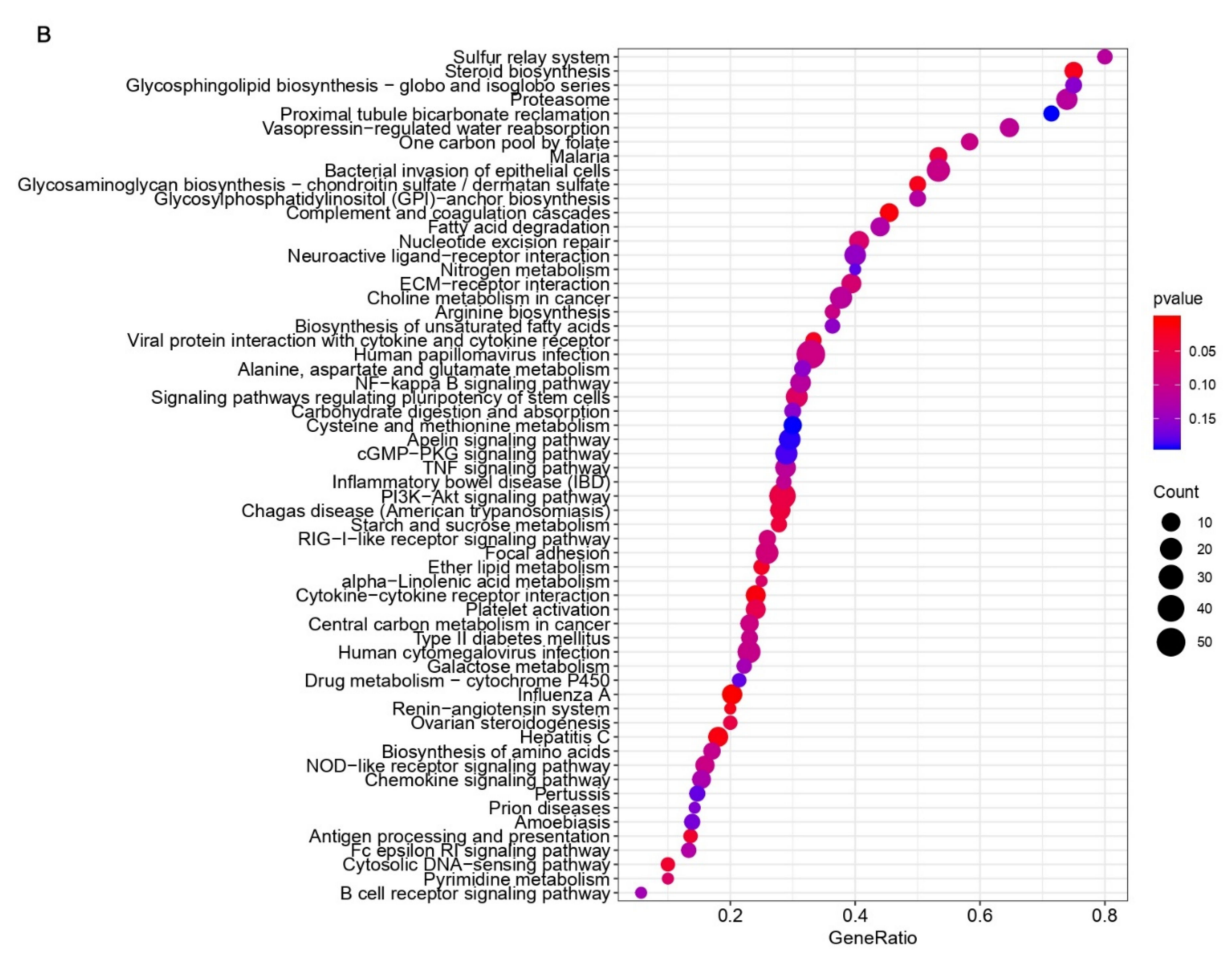
Kyoto Encyclopedia of Genes and Genomes (KEGG) gene set enrichment analysis of DEGs in human (**A**) and pig (**B**) cells at 24 hpi. Enriched signaling pathways with *p* < 0.05 were considered to be statistically significant.

**Figure 6:**
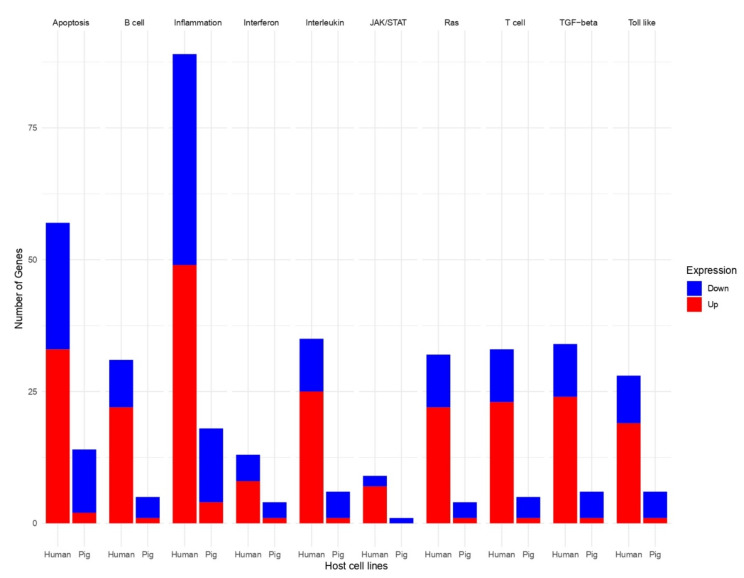
DEGs in 10 common immune-response-associated pathways in pigs and humans during PDCoV infection. Results are shown in 10 pathways: apoptosis signaling pathway, B-cell activation, inflammation/cytokine signaling pathway, interferon signaling pathway, interleukin signaling pathway, JAK-STAT signaling pathway, Ras signaling pathway, T-cell activation, TGF-β signaling pathway, and toll-like receptor signaling pathway. Blue, down-regulated; red, up-regulated.

To identify pathways that were altered upon infection, DEGs were submitted to a gene set enrichment analysis (GSEA) using Kyoto Encyclopedia of Genes and Genomes (KEGG) pathway enrichment. A total of 60 pathways, including 13 immune-response-associated pathways, were enriched in human and pig cells at 24 hpi (Figure 5). From these immune-response-associated pathways, eight were enriched in both cell lines. These included viral protein interaction with cytokine and cytokine receptor, cytokine–cytokine receptor interaction, NF-Kappa-B signaling pathway, B-cell receptor signaling pathway, JAK-STAT signaling pathway, Influenza A associated, toll-like receptor signaling pathway, TNF signaling pathway, NOD-like receptor signaling pathway, and cytosolic DNA-sensing pathway (Figure 5). Additionally, pathways such as the apoptosis signaling pathway, T-cell activation, interferon signaling pathway, interleukin signaling pathway, TGF-β signaling pathway, and Ras signaling pathway were affected in HIEC and IPEC cells after PDCoV infection (Figure 6). From these pathways, there were more genes that were affected in the inflammation/cytokine signaling pathway in pig and human cells in comparison to the other nine pathways (Figure 6). In the same manner, more genes were up-regulated in human and down-regulated in pig cells across the 10 immune-response-associated pathways in response to PDCoV infection (Figure 6).

This study also identified common up-regulated and down-regulated DEGs in human and pig cells across the 10 pathways as well as orthologs between these species (Figures S3 and S4; Tables S3 and S4). Most of the common DEGs that were up-regulated across the 10 pathways are part of the NF-kappa-B transcription factor family (NFKBIA, NFKBIA), interferon (IFNs) family (IFNB1, IFNL1, IFNL3), JAK-STAT family (JAK1, JAK2, STAT1, STAT2), interleukin family (CXCL8), protein-kinase families such as MAPK kinase (MAP3K4, MAPK7) and RAF kinase (RAF1) (Figure S3, Table S3). The interferon family, including MX1 and some other DEGs, were also up-regulated in different categories according to the gene ontology classification (Table S5). By contrast, common DEGs that were down-regulated across the 10 pathways belong to the protein-kinase family such as PIK3CB/LOC100622663, SOS2 and MAPK family (MAPK3, MAPK14) and the FOS family of transcription factors (FOS) (Figure S4, Table S4). There were approximately 16 orthologs of up-regulated and down-regulated DEGs between human and pig cells that were identified in different pathways such as apoptosis signaling pathway, B- and T-cell activation, inflammation/cytokine signaling pathway, interleukin signaling pathway, toll-like receptor signaling pathway, and Ras signaling pathway. From these pathways, five orthologous DEGs were up-regulated in humans cells and down-regulated in pig cells in the apoptosis signaling pathway (MAP4K4 (97.34%), REL (80.55%), NFKB2 (92.78%), BCL2L1 (31.25%), FOS (96.34%)); three orthologous DEGs were up-regulated in human and pig cells in inflammation/cytokine signaling pathway (CXCL8 (77.32%), NFKB2 (92.78%), NFKBIE (86.46%)), and two orthologous DEGs were down-regulated in human and pig cells in the Ras signaling pathway (PIK3CB (97.66%), PLD1 (93.11%)). MAP4K4 was unique in up-regulated DEGs in humans in the apoptosis signaling pathway (Figure S5, Tables S3 and S4).

## 4. Discussion

PDCoV is enteropathogenic and infects villous epithelial cells of the small and large intestines although the jejunum and ileum are the primary sites of infection [24]. Cell lines derived from intestinal epithelial sources recapitulating the sites of primary infection are a relevant in vitro model for studying host response to this viral pathogen. IPEC-J2 cells are intestinal porcine enterocytes that were isolated from the jejunum of a neonatal unsuckled piglet [40]. Jung and colleagues reported that IPEC-J2 cells are susceptible to infection with PDCoV [41]. In this study, we showed, for the first time, that HIEC cells are also susceptible to PDCoV infection (Figure 1). By comparing the global gene expression of IPEC and HIEC cells with and without PDCoV infection, we identified more DEGs in human in comparison to pig cells, and as a result, significantly more genes were up-regulated in human than in pig cells at 24 hpi (Figure 2, Figure 4 and Figure 6; Table 2). We also found that the top 10 up-regulated genes in human cells exhibited a higher magnitude of response upon PDCoV infection (more than a 10-fold change in transcriptional response) compared to the host-related response of pig cells (more than 2.7-fold change) (Tables S2, S7, and S8). From these genes, interferon β-1_Human (IFNB1), interferon-induced protein 44-like_Human (IFI44L), interferon-induced GTP-binding protein, porcine (Mx1), and radical S-adenosyl methionine domain containing two (RSAD2) showed the highest logFC (Tables S2, S7, and S8). These results suggest that at the transcriptional level, human cells show more pronounced and up-regulated responses to PDCoV infection in comparison to pig cells. We speculate that one of the main reasons for the difference in gene expression being higher in human cells than in pig cells is because PDCoV may already be well adapted to the porcine host, while the human host could be a new host for PDCoV. It has been shown that primary human intestinal epithelial cells (HIEC) elicit robust replication of SARS-CoV-2, and the secretion of de novo infectious virus particles is controlled where interferon (IFN) mediated responses are robust [55]. Interestingly, HIEC cells are supporting PDCoV infection by showing a robust intrinsic immune response where pathways related to innate-associated immune response were affected. In fact, virus titers reveal a 10-log increase in viral load (10 Log10) in HIEC cells in comparison to IPEC cells, which can indicate potential virus replication and explain the difference in gene expression between human and pig cells (Figure S2). Nevertheless, the difference in gene expression in both human and pig cell lines could be attributed to a potentially confounding factor such as not being able to control virus-induced cell death rate, the limited database annotation for pigs, and/or difficulties in quantitatively determining the true number of infected cells in both cell types. Future work needs to explore other next-generation sequencing technologies such as single-cell RNA sequencing to address these experimental limitations.

When cells are exposed to pathogens such as viruses, immune responses are induced as a host defense [31]. Similarly, apoptosis is induced as one of the host antiviral responses in order to limit virus replication and production during viral infections [31]. In this study, we identified common pathways and genes related to the innate-associated immune response that were activated after PDCoV infection in human and pig cells. Our data suggest that eight immune-associated signaling pathways were commonly enriched in human and pig cells in response to PDCoV infection (Figure 5). In fact, some of these pathways—including NOD-like receptor signaling pathway, JAK-STAT signaling pathway, the cytosolic DNA-sensing pathways and toll-like receptor signaling pathway—play a pivotal role in innate immune responses [28,56]. With reference to this last signaling pathway, toll-like receptors are the best studied pattern-recognition receptors (PRRs), and to date, 10 functional TLRs are known in human and swine. Studies suggest that IPEC-J2 cells have expressed TLR2, TLR4, and TLR9 and are a valuable tool for the study of these porcine TLRs [56,57]. These pathways perform an important function in the process how the interferon family—specifically type-I IFN—develops antiviral function in the innate immune response [28,56]. Additionally, we found that pathways such as the apoptosis signaling pathway, T-cell activation, interferon signaling pathway, interleukin signaling pathway, TGF-β signaling pathway, and Ras signaling pathway were affected in HIEC and IPEC during PDCoV infection (Figure 6, Figures S6–S11). Most of the DEGs in these pathways were significantly up-regulated in human cells and significantly down-regulated in pig cells at 24 hpi in comparison to cells without PDCoV infection (Figure 6, Figures S6–S11). These results demonstrate that the pathways affected during PDCoV infection can either enhance or inhibit immune responses in IPEC and HIEC cells, suggesting that human cells respond differently to PDCoV infection in comparison to pig cells.

An in-depth analysis showed that, in both cell lines, there are more genes affected in inflammation/cytokine signaling pathway compared to the other nine immune-related pathways (Figure 6). Transcriptome studies related to NK cells have identified expression of interferons (IFNs), tumor-necrosis factor (TNF), and inflammation/cytokine stimulation in the activation of NK cells by viruses [58,59]. Interestingly, these cells can increase the IFNs up to 100-fold [60]. We identified similar responses in HIEC cells infected by PDCoV. Inflammation responses appeared to increase faster in HIEC cells than in IPEC cells (data not shown) suggesting that human cells such as HIEC cells may limit viral replication by efficiently releasing cytokines. However, further studies are needed to determine if this is a host antiviral state for preventing or limiting viral replication or a complication induced by the virus in some individuals, in which in vivo is associated with a cytokine storm that can lead to death in humans such as after SARS-CoV-2 infections [55,61].

It has been demonstrated that the IFNs can be induced by a number of stimuli including viruses and dsRNA through mechanisms that involve the activation of NF-κB [60]. After secretion from the cells, cytokines bind to specific cell-surface receptors and start the induction of several responsive genes via signaling through the JAK-STAT signaling pathway [59]. The regulation of IFNs via this pathway leads to the phosphorylation of STAT1 and STAT 2 and the recruitment of JAK1 and JAK2, which are genes that were up-regulated in human cells in this study and common in some pathways (Table S3 and Figures S10 and S11). Genes of the JAK/STAT family may be required for the optimal expression of several pro-apoptotic genes, suggesting that these genes may have multiple roles in the cell [60].

This study also reveals that there are some common and unique up-regulated and down-regulated DEGs in human and pig cells across 10 pathways as well as orthologs between these species (Tables S3 and S4 and Figures S3–S5). Most of the common DEGs that were up-regulated across the 10 pathways in human and pig cells are part of the NF-kappa-B transcription-factor family, interferon (IFN) family (Figure S3, Table S3), JAK-STAT family (Figures S10 and S11, Table S3) and protein-kinase family such as MAPK kinase and RAF kinase (Table S3); while most of the genes that were down-regulated across the 10 pathways in human and pig cells belong to the protein-kinase family such as the PIK3CB, SOS2, and MAPK family, and the FOS family of transcription factors (Figure S4, Table S4). MAP4K4 was unique in up-regulated DEGs in humans in the apoptosis signaling pathway (Figure S5). MAP4K4 have been involved in focal adhesion dynamics regulation [62], systemic inflammation [63], lung inflammation [64], type 2 diabetes [65], atherosclerosis [66], insulin sensitivity [67], and cancer [68]. These results indicate that these DEGs play an important conserved role in the PDCoV mechanism of infection in human and pig cells.

Previous studies have demonstrated that PDCoV infection fails to induce IFN-β production in LLC-PK1 cells [61]. In our study, we found that IFN-β and INF-λ production in HIEC cells was increased at 24 hpi (Tables S2 and S3), while IFN-γ production (specifically interferon gamma receptor 2 (IFNGR2)) was increased in IPEC cells and reduced in HIEC cells at 24 hpi (Tables S3 and S4). We believe that these responses of the host to the virus are explained by the fact that (1) infection of primary HIEC cells induces a robust intrinsic immune response that can be controlled by type I and type III interferons [55], (2) porcine epithelial cells exhibit a strong response to viruses and other pathogens with production of TNF-α and IFN type I (α and β) and III (λ) which impede pathogen replication [56]; (3) Type III IFNs have a unique tropism where their signaling and functions are restricted to epithelial cells [69], and (4) Type-I IFN response might be induced at 24 hpi and inhibited at 36 hpi by PDCoV in pig kidney (PK-15) cells [28]. Additionally, in humans there are multiple forms of IFN-α, only one type of IFN-β and additional isotypes such as IFN-ĸ, IFN-δ, IFN-ε, IFN-ω, and IFN-τ; while porcine IFN complex consists of both cross-species comparable and specific–specific antiviral IFN subtypes, such as IFN-α/β and IFN-δ, respectively [70]. Interestingly, this study suggests that the production of INF type II (IFN-γ) was affected during PDCoV infection in HIEC and IPEC cells. IFN-γ has several immunoregulatory functions that include optimizing the antiviral response and limiting excessive responses that could lead to damage [71]. It has been shown that in HSV-2 infection, the absence of IFN-γ production can result in increased virus replication and decreases survival [72,73]. In this particular case, we speculate that the decreased production of this interferon in HIEC cells is contributing to the increased virus replication and to the decreased cell survival. It has also shown the secretion of the pro-inflammatory cytokine interferon gamma (IFN-γ) in pregnant pigs. More infection times need to be carefully addressed in future experiments to evaluate if different types of cytokines and interferons are induced by PDCoV in different hosts.

There is growing evidence that IFNs can be activated by the mitogen-activated protein-kinase (MAPK) pathway, and it has been determined recently that MAPKs are important for type III, but not type I, IFN in mediating antiviral protection in human intestinal epithelial cells [69]. In this study, we also identified several up-regulated and down-regulated DEGs that belong to the MAPK-kinase family (Tables S3 and S4). Further in-depth studies are needed to address these results to investigate whether PDCoV infection is characterized by the production of a specific type of interferon in HIEC and IPEC cells.

Finally, this study also identified five orthologous DEGs that were up-regulated in human cells and down-regulated in pig cells in the apoptosis signaling pathway (Tables S3 and S4; Figure S5). An explanation for this can be related to apoptotic cell death induced by a virus which has a complex role in host defense, promotes the clearance of viruses, and/or serves as a mechanism for virus-induced tissue damage and progression of disease [74]. Under these circumstances, we hypothesize that HIEC cells are modifying expression of these specific DEGs to limit PDCoV replication and production. PDCoV is also attempting to reduce the activation of the same set of DEGs and using this as a host innate-immune evasion strategy to be successful in the progression of PDCoV infection in IPEC cells. Of note, three orthologous DEGs were up-regulated in human and pig cells in the inflammation/cytokine signaling pathway, and two orthologous DEGs were down-regulated in human and pig cells in Ras signaling pathway (Figure S5). Together, these results suggest that there are similar aspects in the immune-associated response to PDCoV infection in human and pig cells.

## 5. Conclusions

In summary, we compared the transcriptome of HIEC and IPEC cells after PDCoV infection to model a potential zoonotic host response to an emerging novel pathogen and examined reads, genes, and pathways. To the best of our knowledge, this is the first report of transcriptome analysis of a human intestinal cell line infected by PDCoV. Our results reveal that there are more differentially expressed genes in human compared to pig cells. At the transcriptional level, human cells exhibited a stronger response with more up-regulated DEGs to PDCoV infection compared to pig cells, where interferon (IFN) can play an important role in controlling PDCoV replication and virus production. We also demonstrated key immune-associated DEGs and signaling pathways in response to PDCoV infection that are shared and unique between the cell lines from two host species. MAP4K4 was unique in up-regulated DEGs in humans in the apoptosis signaling pathway. These data provide an important foundation that will contribute to an understanding of the mechanisms of CoV cross-species transmission. Further work is necessary to address if (1) there are more key immune-associated DEGs at other time times of PDCoV infection and (2) there is a different transcriptional response of the virus to other hosts (cell lines) and in vivo systems.

## Figures and Tables

**Table 1:** RNA sequencing and genome mapping results.

Sample	Raw Reads	Clean Reads	Total Mapping (%)
Hs_1	47007321	45421159	95.83%
Hs_2	50022577	48880712	95.52%
Hs_3	43496780	42168797	94.63%
Hs_Dc_1	15600403	14820248	85.82%
Hs_Dc_2	24140872	23537480	82.23%
Hs_Dc_3	48513411	47055941	81.59%
Ss_1	23545237	23195923	94.46%
Ss_2	53210729	51875151	95.94%
Ss_3	53026614	52011848	94.54%
Ss_Dc_1	44124431	42370051	89.67%
Ss_Dc_2	53543270	52298369	86.84%
Ss_Dc_3	50508987	48861970	94.36%

Hs, Homo sapiens; Hs_Dc, Homo sapiens_Deltacoronavirus; Ss, Sus scrofa; Ss_Dc, Sus scrofa_Deltacoronavirus.

**Table 2:** DEGs in *H. sapiens* (HIEC cells) and *S. scrofa* (IPEC cells) at 24 hpi vs no infected cells.

Species	Up	Down	Non.sigf	Total Genes	Total DEGs
*Homo sapiens*	4011	3475	10784	18270	7486
*Sus scrofa*	542	592	10716	11850	1134

Up, upregulated genes; Down, downregulated genes; Non.sigf, genes with no significant differences.

**Table 3:** Validation of differential expression between RT-qPCR and RNA-seq by selected transcripts *.

Gene	Cell Type	RT-qPCR	RNA-seq
***HDHD2***	HIEC	−0.8	−2.3
***KLF2***	HIEC	3.89	1.68
***CATSPER1***	IPEC	−0.13	−1.21
***HSP70.2***	IPEC	−0.29	−1.48
***NEK6***	IPEC	1	1.36
***MX1***	IPEC	2.37	2.04
***RSAD2***	IPEC	2.73	2.63

* The mean fold changes for each group were compared in the table chart for seven transcripts (*HDHD2* (Haloacid dehalogenase like hydrolase domain containing 2); *KLF2* (Kruppel like factor 2); *CATSPER1* (Cation channel sperm associated 1); *HSP70.2* (Heat shock protein 70.2); *NEK6* (NIMA related kinase 6); *MX1* (Interferon-induced GTP-binding protein Mx1); *RSAD2* (Radical S-adenosyl methionine domain containing 2)). RT-qPCR data were normalized to GAPDH expression for each sample. Means of significant (*p* < 0.05) fold changes from control were computed for RT-qPCR, and RNA-seq using RNA from the same cell samples in each analysis.

## Data Availability

The data supporting this study is available in NCBI-SRA (Bioproject No. PRJNA690955) and the computing code is available in GitHub (https://github.com/Diana-Ouma/Comparative-transcriptome-analysis (accessed on 30 December 2020)).

## Supplementary Materials

The following are available online at https://www.mdpi.com/1999-4915/13/2/292/s1, Table S1. Sequences of primers used in qRT-PCR analyses; Table S2. Top 20 differential-expressed genes in human and pig cell lines; Table S3. Common up-regulated DEGs in human and pig cell lines at 24 hpi; Table S4. Common down-regulated DEGs in human and pig cell lines at 24 hpi; Table S5. Gene ontology classification of up-regulated DEGs in human and pig cell lines at 24 hpi; Table S6. Gene ontology classification of down-regulated DEGs in human and pig cell lines at 24 hpi; Table S7. Dataset of DEGs in HIEC cells (See Excel file); Table S8. Dataset of DEGs in IPEC cells (See Excel file). Figure S1. IF staining of the inoculated IPEC cells at 24 hpi. Images taken at 300x total magnification; Figure S2. Viral titers, 24 hpi. Figure S3. Common up-regulated DEGs in human and pig cell lines at 24 hpi across 10 pathways; Figure S4. Common down-regulated DEGs in human and pig cell lines at 24 hpi across 10 pathways; Figure S5. Orthologs of up-regulated and down-regulated DEGs between human and pig cell lines in: Apoptosis signaling pathway, B- and T-cell activation, inflammation/cytokine signaling pathway, interleukin signaling pathway, toll-like receptor signaling pathway, and Ras signaling pathway; Figure S6. Up-regulated and down-regulated DEGs in humans_ cytokine–cytokine receptor interaction pathway; Figure S7. Up-regulated and down-regulated DEGs in pigs_ cytokine–cytokine receptor interaction pathway; Figure S8. Up-regulated and down-regulated DEGs in humans_ apoptosis signaling pathway; Figure S9. Up-regulated and down-regulated DEGs in pigs_ apoptosis signaling pathway; Figure S10. Up-regulated and down-regulated DEGs in humans_ JAK-STAT signaling pathway; Figure S11. Up-regulated and down-regulated DEGs in pigs_ JAK-STAT signaling pathway.
