# Supplementary material for: Comparative Transcriptome Profiling of Human and Pig Intestinal Epithelial Cells after Porcine Deltacoronavirus Infection
Source: Viruses. 2021 Feb 13;13(2):292. doi: 10.3390/v13020292 (PMC7918119; doi:10.3390/v13020292)
Supplement: Supplementary file 1 [file viruses-13-00292-s001.zip › viruses-1078580-suppl/Supp_Inf_Comparative_Transcript spk.doc]

**Supporting Information**

**Table S1.** Sequences of primers used in qRT-PCR analyses.

| **Gene** | **Oligonucleotide Sequence (5’→3’)** | **Size (bp)** |
| --- | --- | --- |
| **GAPDH** | ACC CAG AAG ACT GTG GAT GG (F) | 124 |
|  | TCA GCT CAG GGA TGA CCT TG ( R ) |  |
| **CATSPER1** | GAG CAT CCT CAC CAC CAT CT (F) | 310 |
|  | GTG TTC GCT CAT CAC CTC CT (R) |  |
| **HDHD2** | CCA GAA GTT TAC TAG AGC GGA AA (F) | 114 |
|  | ATC CCA TGA CCA CAG CAT TAG ( R ) |  |
| **HSP70.2** | CGA TGG TGC TGA CCA AGA TG (F) | 102 |
|  | GCT GCG AGT CGT TGA AGT AG ( R ) |  |
| **KLF2** | ACC TAC ACC AAG AGT TCG CA (F) | 167 |
|  | CAC AGA TGG CAC TGG AAT GG ( R ) |  |
| **MX1** | CCA CCT GAA GGG CTA CA (F) | 139 |
|  | CCC TTC CTC CAG AAG ATC CC ( R ) |  |
| **NEK6** | GAC AGG AAG ACA GTG GCT CT (F) | 165 |
|  | CAC GAT GTT CAG CTC GTT GT ( R ) |  |
| **RSAD2** | CTT AGA CCG CCA CAA AGA CG (F) | 114 |
|  | CGC CCG TTT CTA CAG TTC AG ( R ) |  |

**Table S2. Top 20 differential expressed genes in human and pig cell lines**

| **Ensembl ID** | **Gene Symbol** | **Species** | **logFC** | **logCPM** | **P-value** | **FDR** | **Expression** |
| --- | --- | --- | --- | --- | --- | --- | --- |
| **ENSG00000171855** | IFNB1 | *Homo sapiens* | 10.58987 | 1.872253 | 3.75E-109 | 3.43E-106 | Up |
| **ENSG00000137959** | IFI44L | *Homo sapiens* | 7.578162 | 5.152949 | 1.81E-148 | 4.73E-145 | Up |
| **ENSG00000182393** | IFNL1 | *Homo sapiens* | 6.661177 | 0.255202 | 1.02E-36 | 1.23E-34 | Up |
| **ENSG00000089127** | OAS1 | *Homo sapiens* | 6.056076 | 3.024683 | 7.22E-99 | 5.49E-96 | Up |
| **ENSG00000197110** | IFNL3 | *Homo sapiens* | 6.04519 | -2.12294 | 4.45E-08 | 6.04E-07 | Up |
| **ENSG00000160505** | NLRP4 | *Homo sapiens* | 6.964062 | -1.43834 | 3.24E-12 | 8.50E-11 | Up |
| **ENSG00000171560** | FGA | *Homo sapiens* | 6.937664 | -1.46837 | 6.65E-11 | 1.49E-09 | Up |
| **ENSG00000171557** | FGG | *Homo sapiens* | 9.368923 | 0.642956 | 4.41E-48 | 7.67E-46 | Up |
| **ENSG00000122025** | FLT3 | *Homo sapiens* | 5.550694 | 1.443451 | 1.18E-49 | 2.17E-47 | Up |
| **ENSG00000146151** | HMGCLL1 | *Homo sapiens* | -1.30409 | 1.70339 | 6.21E-07 | 6.71E-06 | Down |
| **ENSG00000241399** | CD302 | *Homo sapiens* | -1.2697 | 1.857402 | 1.16E-05 | 9.19E-05 | Down |
| **ENSG00000188037** | CLCN1 | *Homo sapiens* | -1.30207 | -1.21937 | 0.022515 | 0.061942 | Down |
| **ENSG00000111775** | COX6A1 | *Homo sapiens* | -2.15232 | -1.40514 | 0.00119 | 0.005294 | Down |
| **ENSG00000115290** | GRB14 | *Homo sapiens* | -1.29817 | 0.132904 | 0.000525 | 0.002599 | Down |
| **ENSG00000149084** | HSD17B12 | *Homo sapiens* | -1.66029 | -0.77379 | 0.007978 | 0.026197 | Down |
| **ENSG00000136542** | GALNT5 | *Homo sapiens* | -1.64265 | 0.786164 | 9.36E-07 | 9.72E-06 | Down |
| **ENSG00000145757** | SPATA9 | *Homo sapiens* | -2.11211 | -1.72707 | 0.005062 | 0.018026 | Down |
| **ENSG00000124678** | TCP11 | *Homo sapiens* | -2.03175 | -1.04844 | 0.000526 | 0.002602 | Down |
| **ENSG00000169435** | RASSF6 | *Homo sapiens* | -1.61692 | -0.66667 | 0.001183 | 0.00527 | Down |
| **ENSG00000112175** | BMP5 | *Homo sapiens* | -1.83933 | 0.846557 | 6.13E-06 | 5.24E-05 | Down |
| **ENSSSCG00000012077** | MX1 | *Sus scrofa* | 2.041311 | 2.158071 | 8.89E-06 | 0.005018 | Up |
| **ENSSSCG00000008648** | RSAD2 | *Sus scrofa* | 2.635093 | 0.56878 | 2.12E-07 | 0.000419 | Up |
| **ENSSSCG00000017705** | CCL5 | *Sus scrofa* | 1.952532 | -0.72996 | 0.00204 | 0.137896 | Up |
| **ENSSSCG00000007682** | SH2B2 | *Sus scrofa* | 1.144246 | -0.04913 | 0.013264 | 0.335123 | Up |
| **ENSSSCG00000016850** | LIFR | *Sus scrofa* | 1.289662 | -0.93357 | 0.047963 | 0.516218 | Up |
| **ENSSSCG00000005587** | NEK6 | *Sus scrofa* | 1.359133 | -0.15415 | 0.04673 | 0.510582 | Up |
| **ENSSSCG00000012520** | BHLHB9 | *Sus scrofa* | 1.406445 | -0.07198 | 0.00568 | 0.233698 | Up |
| **ENSSSCG00000017416** | DHX58 | *Sus scrofa* | 1.140561 | 0.216966 | 0.012688 | 0.32265 | Up |
| **ENSSSCG00000005311** | CD72 | *Sus scrofa* | -1.83387 | -0.28071 | 0.000267 | 0.046614 | Down |
| **ENSSSCG00000012959** | CATSPER1 | *Sus scrofa* | -1.21347 | -0.30285 | 0.032173 | 0.464939 | Down |
| **ENSSSCG00000020740** | DSG4 | *Sus scrofa* | -1.43382 | -0.29562 | 0.001842 | 0.133961 | Down |
| **ENSSSCG00000030368** | HSP70.2 | *Sus scrofa* | -1.47794 | -0.58141 | 0.015439 | 0.353103 | Down |
| **ENSSSCG00000027997** | NME5 | *Sus scrofa* | -1.42071 | -0.45481 | 0.005247 | 0.222052 | Down |
| **ENSSSCG00000030850** | TEX11 | *Sus scrofa* | -1.23861 | -0.81813 | 0.042827 | 0.497553 | Down |
| **ENSSSCG00000006648** | CTSS | *Sus scrofa* | -1.71573 | -0.60694 | 0.002659 | 0.158376 | Down |

**Table S3.** Common upregulated DEGs in human and pig cell lines at 24 hpi

*Common genes across pathways and between humans and pigs are highlighted in red.

**Table S4.** Common downregulated DEGs in human and pig cell lines at 24 hpi

*Common genes across pathways and between humans and pigs are highlighted in blue.

**Table S5.** Gene Ontology classification of upregulated DEGs in human and pig cell lines at 24 hpi

| **Category** | **Description** | **Human** | **Pig** |
| --- | --- | --- | --- |
| **BP** | Antiviral Defense, Influenza A | IFNB1, IFI44L, IFNL1, OAS1, IFNL3 | MX1, RSAD2 |
| **BP** | Defense response to virus | IFNB1, IFI44L, IFNL1, OAS1, IFNL3 | CCL5,MX1,RSAD2 |
| **BP** | Negative regulation of viral genome replication | IFNB1, OAS1, IFNL3 | CCL5, MX1, RSAD2 |
| **BP** | Cytokine and Inflammatory response | IFNB1, IFNL1, IFNL3, NLRP4 | CCL5, SH2B2, RSAD2 |
| **MF** | Cytokine activity | IFNB1, IFNL1, IFNL3 | LIFR |
| **KEGG_PATHWAY** | Jak-STAT signaling pathway | IFNB1, IFNL1, IFNL3 | LIFR |
| **KEGG_PATHWAY** | Cytokine-cytokine receptor interaction | IFNB1, IFNL1, IFNL3 | LIFR, CCL5 |
| **BP** | Innate immune response | FGA, IFNL1, IFNL3 | MX1, RSAD2 |
| **BP** | Pos/Neg Regulation of endothelial cell apoptotic process | FGA, FGG | CCL5, NEK6, BHLHB9 |
| **BP** | Pos/Neg regulation of apoptotic signaling via death domain | FGA, FGG | CCL5, NEK6, BHLHB9 |
| **BP** | B cell differentiation | IFNB1, FLT3 | SH2B2 |
| **BP** | Type I interferon signaling pathway | IFNB1, OAS1, NLRP4 | CCL5, DHX58, MX1 |
| **BP** | negative regulation of T cell differentiation | IFNB1, IFNL1 | CCL5 |

*Common genes across pathways and between humans and pigs are highlighted in red.

**Table S6.** Gene Ontology classification of downregulated DEGs in human and pig cell lines at 24 hpi .

|  |  | **Gene Name** | |
| --- | --- | --- | --- |
| **Category** | **Description** | **Human** | **Pig** |
| **CC** | Membrane | HMGCLL1, CD302, CLCN1,COX6A1, GRB14, HSD17B12, GALNT5, SPATA9, TCP11 | CD72, CATSPER1, DSG4 |
|  |  |  |  |
| **BP** | Estrogen biosynthetic process | HSD17B12 | HSP70.2 |
| **BP** | steroid biosynthetic process | HSD17B12 | HSP70.2 |
| **BP** | Ras-association and Apoptotic process | RASSF6, GRB14, BMP5, STAR | NME5, TEX11 |
| **KEGG_PATHWAY** | Antigen processing and presentation | CD302 | CTSS, HSP70.2 |

*Common genes across pathways and between humans and pigs are highlighted in blue.

**Table S7.** Dataset of DEGs in HIEC cells (See Excel file).

**Table S8.** Dataset of DEGs in IPEC cells (See Excel file).

**Figure S1. IF staining of the inoculated IPEC cells at 24 hpi.** A) IF stained positive (green fluorescence) for PDCoV antigen. B) Blue-fluorescent 4’,6-diamidino-2-phenylindole dihydrochloride (DAPI) staining of Panel A to stain nuclear DNA. C) Overlay of panels A and B. The arrowheads indicated clustered of cells positive for PDCoV antigen. D) IF staining of PDCoV-uninoculated (0.05% Trypsin). E) DAPI staining of Panel D. F) Overlay of panels D and E. Images were taken at 300x total magnification.

**PDCoV positive by IF staining**

**DAPI staining of Panel A**

**Overlay of Panels A and B**

**IPEC Negative control**

**DAPI staining of Panel D**

**Overlay of Panels D and E**

D

E

F

A

B

C

**Figure S2. Viral titers, 24hpi.** qRT-PCR was performed on extracted RNA samples in triplicate using PDCoV-specific primers. Viral load is expressed as genomic equivalents per mL. Data was compared using students unpaired t-test. **** P value is <0.0001.

**Figure S3.** Common upregulated DEGs in human and pig cell lines at 24 hpi across 10 pathways.

**Figure S4.** Common downregulated DEGs in human and pig cell lines at 24 hpi across 10 pathways.

**Figure S5.** Orthologs of upregulated and downregulated DEGs between human and pig cell lines in: Apoptosis signaling pathway, B and T cell activation, Inflammation/cytokine signaling pathway, Interleukin signaling pathway, Toll like receptor signaling pathway and Ras signaling pathway.

***** Up_H, Upregulate_Humans; Down_H, Downregulate_Humans; Up_P, Upregulate_Pigs; Down_P, Downregulate_Pigs.

**Figure S6.** Upregulated and downregulated DEGs in humans_ Cytokine – Cytokine Receptor Interaction Pathway

**Figure S7.** Upregulated and downregulated DEGs in pigs_ Cytokine – Cytokine Receptor Interaction Pathway

**Figure S8.** Upregulated and downregulated DEGs in humans_ Apoptosis signaling pathway

**Figure S9.** Upregulated and downregulated DEGs in pigs_ Apoptosis signaling pathway

**Figure S10.** Upregulated and downregulated DEGs in humans_ JAK-STAT signaling pathway

|  | **Number of shared genes** |  |
| --- | --- | --- |
| **Pathway** |  | **Gene name** |
| Down_P Up_H | 1 | FOS |
| Up_H | 1 | NFKB2 |

**Figure S11.** Upregulated and downregulated DEGs in pigs_ JAK-STAT signaling pathway
